# Effects of Chemical and Physical Enhancement Techniques on Transdermal Delivery of Cyanocobalamin (Vitamin B12) *In Vitro*

**DOI:** 10.3390/pharmaceutics3030474

**Published:** 2011-08-10

**Authors:** Ye Yang, Haripriya Kalluri, Ajay K. Banga

**Affiliations:** College of Pharmacy and Health Sciences, Mercer University, Atlanta, GA 30341, USA; E-Mail: yangy_30329@yahoo.com (Y.Y.); haripriya.kalluri@live.mercer.edu (H.K.)

**Keywords:** cyanocobalamin, Vitamin B12, microneedles, iontophoresis, chemical enhancers, transdermal, skin, drug delivery

## Abstract

Vitamin B12 deficiency, which may result in anemia and nerve damage if left untreated, is currently treated by administration of cyanocobalamin via oral or intramuscular routes. However, these routes are associated with absorption and compliance issues which have prompted us to investigate skin as an alternative site of administration. Delivery through skin, however, is restricted to small and moderately lipophilic molecules due to the outermost barrier, the stratum corneum (SC). In this study, we have investigated the effect of different enhancement techniques, chemical enhancers (ethanol, oleic acid, propylene glycol), iontophoresis (anodal iontophoresis) and microneedles (soluble maltose microneedles), which may overcome this barrier and improve cyanocobalamin delivery. Studies with different chemical enhancer formulations indicated that ethanol and oleic acid decreased the lag time while propylene glycol based formulations increased the lag time. The formulation with ethanol (50%), oleic acid (10%) and propylene glycol (40%) showed the maximum improvement in delivery. Iontophoresis and microneedle treatments resulted in enhanced permeation levels compared to passive controls. These enhancement approaches can be explored further to develop alternative treatment regimens.

## 1. Introduction

Vitamin B12 is essential for the development of red blood cells, body growth, and maintenance of the nervous system [1]. Recent surveys suggest that about 1.5 to 15% of the population is affected by Vitamin B12 deficiency [2-5]. It is mainly prevalent in the older generation with a high incidence rate of ∼40% [6], due to changes in their digestive systems; and in strict vegans, as it is available only from natural animal products such as eggs, fish and meat. Patients with pernicious anemia and other intestinal disorders also commonly develop vitamin B12 deficiency because of their inability to absorb small amounts of the vitamin. When left untreated, this may lead to anemia and irreversible nerve damage. Cyanocobalamin, a cyano- form of vitamin B12 which has now become the main mode of treatment for vitamin B12 deficiency, was first isolated in 1948 [7,8]. Treatment for this condition is mainly in the form of oral aids or injections. The oral form of administration has been associated with concerns related to unpredictable absorption profiles [9], and hence is rarely prescribed other than in Sweden and Canada. Most people with vitamin B12 deficiencies are treated in primary care with intramuscular or deep subcutaneous injections, with some patients requiring monthly intramuscular injections for their whole lives. However, intramuscular administration usually requires a special trip to a health facility or a home visit by a health professional to administer the injection, thus making it less patient-compliant. Therefore, a new method of administration that could overcome the problems associated with intramuscular injections and oral administration is very necessary. Alternative routes of administration include transdermal, buccal, rectal and nasal routes. Of these, the transdermal route is more appealing due to high patient compliance, accessibility and large surface area for drug administration. Transdermal Drug Delivery Systems (TDDS) are non-invasive delivery systems that offer several important advantages over other traditional dosage forms such as high patient compliance, avoidance of gastrointestinal drug metabolism and elimination by liver [10]. However, application of transdermal delivery to a wider range of drugs is limited due to the outermost barrier of skin, the stratum corneum. This is especially problematic for relatively large drugs (molecular mass >500 Da) which represent a large majority of active agents for therapeutic applications. Therefore, various approaches have been undertaken to enhance the delivery of drugs through skin. These strategies include passive/active chemical and physical permeation enhancement techniques to bypass the stratum corneum barrier.

Chemical penetration enhancements (CPEs) are present in a large number of transdermal, dermatological, and cosmetic products to aid dermal absorption of curatives and anesthetics. They provide several advantages such as design flexibility with formulation chemistry, patch application over a large area (>10 cm^2^) and the absence of external physical delivery mechanisms. Several different classes of CPEs, including surfactants, fatty acids, fatty esters and azones, have been studied for enhancement of drug permeation [10]. The effects of these compounds vary accordingly with different drug molecules. Iontophoresis, on the other hand, is a physical enhancement technique which uses a low-level electric current to drive ionized and neutral drug molecules into or across the skin, resulting in higher fluxes of drug molecules [11]. It provides the advantage of controlled drug release from a patch by regulating parameters such as current density and duration of current application.

Microneedle technology is a more recent physical enhancement technique which has shown encouraging results for improving transdermal delivery of drugs [12-15]. Needles of micron sized dimensions can pierce the skin surface and create microchannels large enough for the delivery of macromolecules, but small enough to avoid pain or significant damage. *In vitro* and *in vivo* experiments have shown that pretreating skin with microneedles can increase permeability by orders of magnitude for small drugs and large macromolecules [16]. Since the created microchannels are micron-sized in dimensions, there is no size restriction on drugs that can be delivered by this technology, enabling even the delivery of nanoparticles and microparticles [17].

In this study, we have evaluated the permeation profiles of cyanocobalamin, *in vitro*, to study the effects of chemical enhancers, iontophoresis and microneedles on its transdermal delivery. In addition to permeation experiments, thermal analysis was carried out by differential scanning calorimetry (DSC) to characterize the effects of chemical enhancement on the stratum corneum.

## 2. Materials and Methods

### 2.1. Materials

Cyanocobalamin, oleic acid (OA), sodium chloride, phosphoric acid, potassium dihydrogen phosphate, ethanol (EtOH) and methanol (High-Performance Liquid Chromatography (HPLC) grade) were purchased from Fisher Scientific (Pittsburgh, PA, USA). Propylene glycol (PG) was purchased from Sigma (St. Louis, MO, USA). Maltose microneedles, 500 μm long, were purchased from Texmac Inc. (Charlotte, NC, USA). Each unit consists of a one line of microneedles with 27 microneedles which dissolve upon insertion into skin.

Male hairless rats (300–400 g), the animal model for these studies, were obtained from Charles River Laboratories (Wilmington, MA, USA), and were housed in the Mercer University animal facility until use. Animals were sacrificed via carbon dioxide asphyxiation. All the animal studies were reviewed and approved by the Mercer University Institutional Animal Care and Use Committee.

### 2.2. Methods

#### 2.2.1. Passive permeation of cyanocobalamin

*In vitro* passive permeation of cyanocobalamin was evaluated using Hanson MicroettePulse™ sampling system (Hanson Research Corporation, Chatsworth, CA). Hairless rats were sacrificed as mentioned earlier and the abdominal skin was excised and carefully cleaned to remove any excess fat. Skin samples were mounted between the receptor and donor chambers with the stratum corneum side in contact with the donor solution. The especially designed donor chamber was filled with 1.3 mL of the donor formulation consisting of 0.5, 1, 5 or 10 mg/mL cyanocobalamin in phosphate buffer. The receptor chamber was filled with 7 mL of phosphate buffer (PBS; pH 7.4) and the temperature was maintained at 37 °C. Samples (500 μL) were collected over a period of 24 h at specified time intervals and the receptor solution was replenished accordingly. Experimental samples were analyzed via HPLC.

#### 2.2.2. Effect of chemical enhancers

The effect of the selected chemical enhancers on the stratum corneum was studied using Differential Scanning Calorimetry (DSC). Epidermis was first separated from full thickness skin and then incubated with 1% trypsin PBS solution with the dermis side facing down for 4 h at 37 °C. The tissue was then smoothed out on a parafilm and the stratum corneum was removed by rubbing with a moistened cotton-tipped applicator. The transparent stratum corneum obtained was briefly floated on water, blotted dry, and then was vacuum dried and stored in a desiccator for 2 days. The effect of the selected chemical enhancers on the SC was then investigated via DSC. Untreated stratum corneum and chemical enhancers (EtOH (50%) + OA (10%) + PG (40%)) treated stratum corneum samples were scanned at 1 °C /min. over a temperature range of 20–110 °C.

To check the effect of chemical enhancers on the permeation of cyanocobalamin, *in vitro* experiments were carried out with different chemical enhancers in the donor compartment. For pretreatment of skin samples with chemical enhancers, skin samples were mounted onto Hanson cells. The receptor compartments were filled with phosphate buffer and the donor compartments were filled with 0.5 mL of formulations as listed in Table 1. After 2.5 h, the skin samples were removed and rinsed with 50% EtOH, water and phosphate buffer. The pretreated skin samples were then employed in *in vitro* studies. The same procedure was carried out as discussed earlier and the experimental samples were analyzed via HPLC.

#### 2.2.3. Iontophoresis mediated transdermal delivery of cyanocobalamin

Iontophoresis, an active enhancement technique, was investigated for enhancing permeation levels of cyanocobalamin across hairless rat skin. *In vitro* studies were performed as described earlier. The donor chamber was filled with 0.5 mL of donor formulation consisting of 10 mg/mL cyanocobalamin. Anodal iontophoresis was performed where the Ag electrode was placed in the donor chamber and the AgCl electrode was placed in the sampling port of the receptor chamber; care was taken to avoid any contact between anode and the skin to avoid high local voltage. Electrodes were connected in series to a constant current source (Keithley 2400 SourceMeter^®^, Keithley Instruments Inc., Cleveland, OH) and a current density of 0.3 mA/cm^2^ was applied for a period of 4 h. Samples (300 (μL) were collected from the sampling arm of the receptor compartment at specified time intervals, over a period of 24 h and the receptor buffer was replenished. The samples were later analyzed by HPLC.

#### 2.2.4. Microneedles mediated transdermal delivery of cyanocobalamin

Maltose microneedles were also investigated as a physical enhancement technique to enhance the permeation levels of cyanocobalamin across skin. For microporation, skin samples were stretched on a flat platform, following which microneedles were manually inserted and held in place for ∼1 min. To confirm creation of microchannels by these microneedles, calcein imaging studies were performed. Immediately following microporation, calcein (a fluorescent dye) was applied on the site for ∼1 min. Excess dye was removed with kimwipes and a fluorescent image was taken to observe the pattern. The imaging equipment consisted of a digital camera fitted with a long pass filter with a wavelength of 525 nm.

To check the effect of microporation on permeation of cyanocobalamin, abdominal skin samples were microporated with two single-line microneedle arrays. The microneedle treated skin samples were then sandwiched between the donor and receptor compartments with the stratum corneum side facing the donor chamber. Permeation studies were carried over a period of 24 h as described earlier. Experimental samples were analyzed via HPLC.

#### 2.2.5. Analysis of experimental samples

Samples from all the *in vitro* permeation studies were analyzed via High Pressure Liquid Chromatography (HPLC). A Waters LC module I system was used with a Luna C18 column (5 μm, 150 × 4.6 mm; Phenomenex, CA, USA). The mobile phase consisted of 0.02 M potassium dihydrogen phosphate solution (pH 3.5) and methanol (67:33). A flow rate of 0.5 mL/min was set with a detection wavelength of 360 nm. The retention time was approximately 5.6 minutes. The cumulative amount of drug permeated per unit area was plotted against time and the steady-state flux (J_ss_) and lag time (LT) were calculated from the slope and *x*-intercept of the linear portion, respectively.

All data are presented as mean ± Standard Error (SE). Statistical analysis of the data obtained pre-and post-treatment were done using Student's unpaired t-test, and the data obtained from different groups was analyzed using ANOVA. The level of significance was set as p ≤ 0.05.

## 3. Results and Discussion

**Figure 1.**
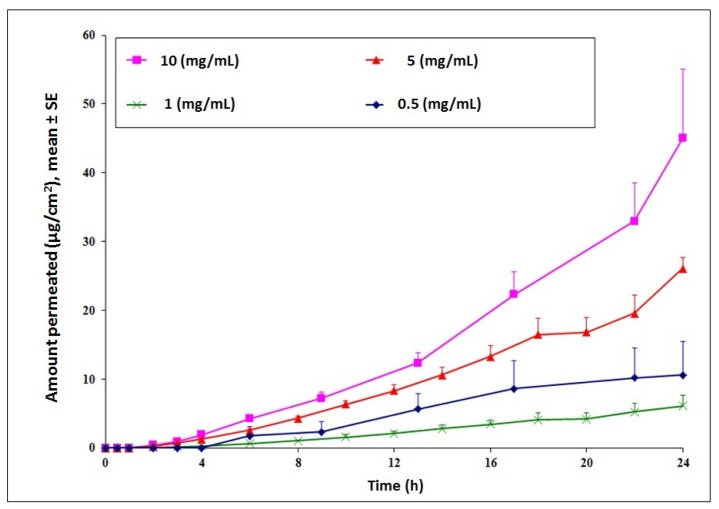
Effect of donor concentration on cyanocobalamin delivery. Passive permeation of cyanocobalamin indicated a concentration dependent delivery with maximum delivery at 10 mg/mL.

Transdermal delivery of cyanocobalamin was investigated to establish feasibility for the long term goal of developing an alternative treatment route for Vitamin B12 deficiency. Initial permeation studies across intact hairless rat skin indicated low permeation of the compound. Cumulative amount delivered at the end of 24 h increased in a concentration dependent manner with a maximum delivery observed for the 10 mg/mL donor concentration (Figure 1). For future studies, to check the effect of chemical and physical enhancement techniques on delivery of cyanocobalamin, the donor concentration was selected as 10 mg/mL.

**Figure 2.**
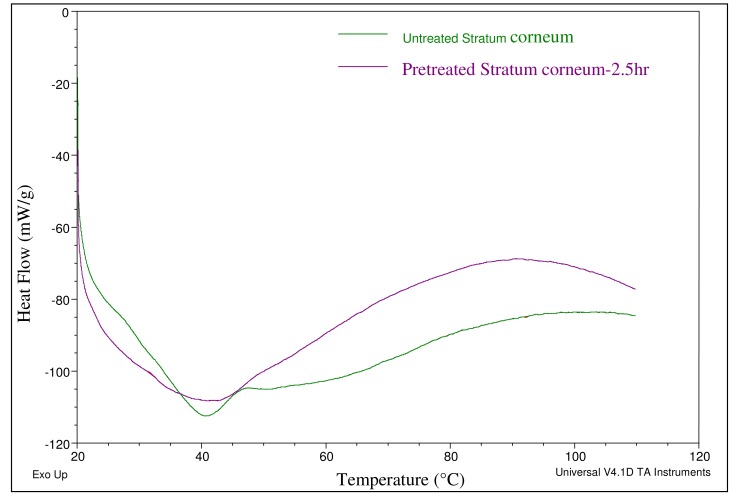
Differential Scanning Calorimetry was performed to study the effect of chemical enhancers (ethanol (EtOH) (50%) + oleic acid (OA) (10%) + Propylene glycol (PG) (40%)) on stratum corneum. The thermogram shows a broadening of the peak at 41 °C which is indicative of disruption of the lipid profile upon treatment with chemical enhancers.

Chemical enhancers were investigated as a means of increasing permeation levels across skin (Table 1). Chemical enhancers typically increase skin permeability by altering the lipid structure of the stratum corneum. Ethanol and oleic acid are commonly used chemical enhancers for transdermal drug delivery where ethanol possesses the dual function of acting as a solvent and a permeation enhancer. The effect of the combination of these two enhancers (EtOH (50%) + OA (10%) + PG (40%) on the lipid profile of the stratum corneum was investigated by Differential Scanning Calorimetry (DSC). Thermal analysis indicated an endothermic peak corresponding to the phase transition of constituent lipids at 41 °C in both pretreated and untreated stratum corneum specimens (Figure 2). However, for the pretreated stratum corneum, a broadening of the endothermic peak was observed which indicated that structures of lipid bilayers were slightly disrupted by the pretreatment. The possibility that this disruption could further aid enhanced delivery across skin was then investigated via *in vitro* permeation studies.

**Figure 3.**
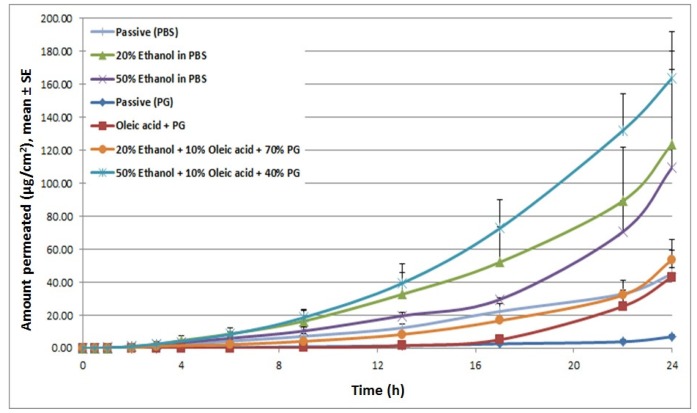
Effect of different chemical enhancers on permeation levels of cyanocobalamin.

*In vitro* permeation studies were then performed to check for the expected increase in drug delivery. The different chemical enhancers and formulations tested have been summarized in Table 1. Cyanocobalamin in phosphate buffered solution (PBS) demonstrated baseline permeability with a cumulative delivery of 45.01 μg/cm^2^, at 24 h, and a lag time of 3.24 h. Compared to the PBS control, the formulation with 20% ethanol increased cyanocobalamin flux by 2-fold. Increasing ethanol concentration even higher, up to 50%, did not show a further enhancement in flux. However, it reduced the lag time from 3.24 h (for PBS alone) to 2.05 h. On the other hand, when propylene glycol (PG) was employed as a vehicle, it reduced the steady-state flux and increased the lag time. The reason for this pattern might be due to dehydration of skin which in turn reduced the thermodynamic activity of cyanocobalamin in the vehicle, mainly due to the high viscosity of PG. Incorporation of 10% oleic acid to the PG formulation increased flux by 3.79-fold as compared with PG control group and reduced the flux by 3-fold as compared with PBS alone. Further addition of ethanol to OA-PG formulation (50% EtOH + 10% OA + 40% PG) enhanced flux by 28.5-fold compared to the PG control and 2.6-fold compared to PBS alone (Figure 3). These permeation profiles confirm our speculations from the DSC data that different chemical enhancers induced different changes in the lipid structure of the stratum corneum which in turn affected cyanocobalamin delivery.

**Figure 4.**
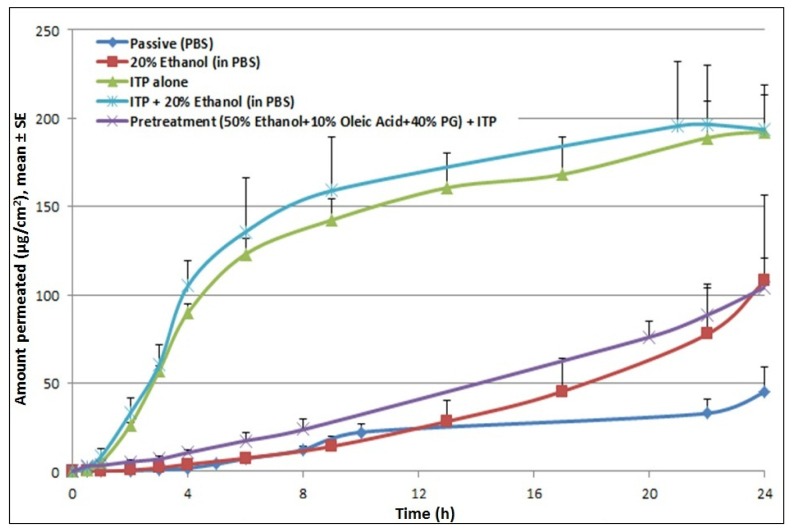
Effect of iontophoresis on transdermal delivery of cyanocobalamin; iontophoresis significantly increased the cumulative amount delivered across skin.

Application of iontophoresis (at 0.3 mA for 4 h) significantly increased permeation levels of cyanocobalamin (27.4 μg/cm^2^/hr) compared to the passive control. Iontophoretic treatment increased the steady state flux by 17-fold and reduced the lag time by 4-fold over passive permeation. Incorporation of a chemical enhancer (20% ethanol) in the donor formulation which was then subjected to iontophoretic treatment resulted in a further increase in permeation compared to the pretreatment control. However, it did not result in any further enhancement compared to ITP alone (Figure 4). Studies were also performed to check the effect of pretreatment with propylene glycol based solutions. Since the propylene glycol based formulation (50% EtOH + 10% OA + 40% PG) cannot be used as a donor vehicle for iontophoresis studies, skin was pretreated with the formulation and permeation study was carried out with a donor formulation containing cyanocobalamin in PBS. The amount of drug delivered was higher than passive control, however very low compared to ITP alone, suggesting no synergistic effects of pretreatment with chemical enhancers and iontophoretic treatment on cyanocobalamin delivery.

These findings are in accord with data reported from another study where iontophoretic treatment with buspirone hydrochloride (BH) formulation containing ethanol/water resulted in a 5-fold reduction in the flux of the compound compared to delivery from BH in water formulation (same concentration and current density) [18]. It was speculated that the addition of ethanol reduced vehicle conductivity and consequently reduced the amount of current carried by the BH ions. Moreover, the addition of ethanol is thought to inhibit the ionization of BH which thereby decreases its specific conductance. Hence, in this study, conductivity of donor solutions was measured. Incorporation of 20% EtOH reduced the conductance of the donor formulation from 16120 μs/cm to 9607 μs/cm compared to the control. This explains the pattern of permeation profiles from our studies where no further enhancement was observed for the combination treatment of chemical enhancers and iontophoresis compared to iontophoresis alone. However, it is to be noted that, for the combination treatment, even though pretreatment did not show a further increase in steady-state flux, after 8 h, the cumulative amount of cyanocobalamin kept increasing until 24 h, while it reached a plateau for iontophoresis alone treatment.

**Figure 5.**
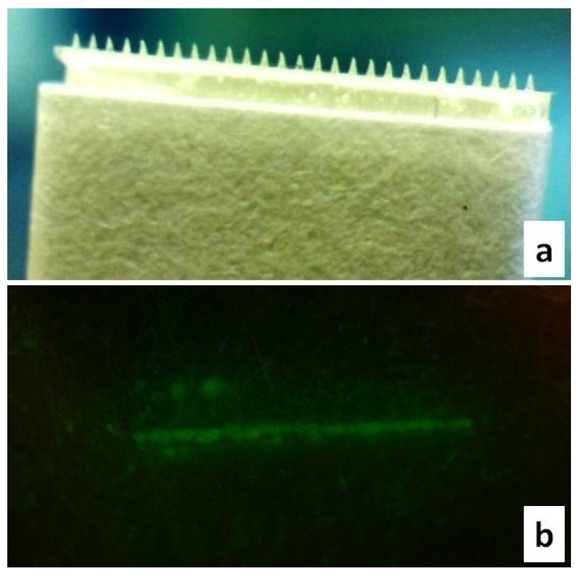
Maltose microneedles: **(a)** Video microscopic image of a single line of maltose microneedles; **(b)** Calcein imaging indicated successful creation of microchannels; single line.

Microneedles were also investigated as an alternative treatment method. Maltose microneedles (Figure 5a) employed in our studies effectively created microchannels as confirmed by calcein imaging studies (Figure 5b). These soluble microneedles have already been extensively characterized for transdermal drug delivery [19-21].

**Figure 6.**
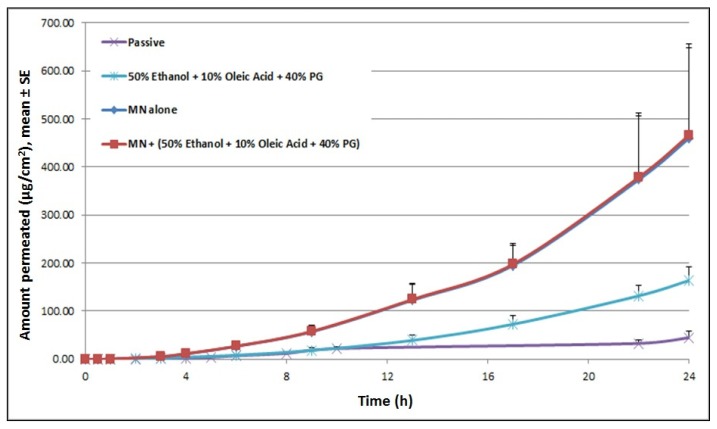
Microneedle mediated transdermal delivery of cyanocobalamin. Drug delivery increased significantly compared to the passive control. Combination with chemical enhancers did not result in a further increase in cyanocobalamin delivery.

Upon treatment with microneedles, steady-state flux of cyanocobalamin increased 13-fold, with an increase in total cumulative amount delivered from 45.01 μg/cm^2^ for passive delivery to 459.69 μg/cm^2^ for MN alone (at 24 h). Pretreating skin with chemical enhancers (50% EtOH + 10% OA + 40% PG), prior to miconeedle mediated delivery did not result in any further enhancement in cyanocobalamin delivery (Figure 6). A phenomenon similar to ITP delivery was observed here. For the combination treatment, even though pretreatment did not show a further increase in steady-state flux, after 8 h, the cumulative amount of cyanocobalamin kept increasing until 24 h, while it reached a plateau for treatment with microneedle alone.

Overall, chemical enhancers, iontophoresis and microneedles increased the steady-state flux of cyanocobalamin significantly over passive delivery. Iontophoresis and microneedle treatments were much more efficient in enhancing permeation levels. Given the advantages associated with these enhancement routes over existing treatment regimens, it is desirable to explore these routes further.

## 4. Conclusions

A transdermal route for delivering cyanocobalamin holds promise when enhancement techniques are incorporated. Therapeutically relevant doses could be delivered through skin with the use of iontophoresis and microneedles. These treatment modes need to be explored further to develop an alternative therapy which will overcome the compliance and absorption issues associated with currently used treatments.

## Figures and Tables

**Table 1.** List of chemical enhancers investigated for enhanced cyanocobalamin delivery.

**Chemical Enhancers**
Phosphate buffer + 20% ethanol
Phosphate buffer + 50% ethanol
10% oleic acid + propylene glycol
20% ethanol + 10% oleic acid + 70% propylene glycol
50% ethanol + 10% oleic acid + 40% propylene glycol
